# Treatment Stratification in First-Line Recurrent or Metastatic Head and Neck Cancer, on Behalf of the EORTC Young Investigator Head and Neck Cancer Group

**DOI:** 10.3389/fonc.2022.730785

**Published:** 2022-01-27

**Authors:** Konrad Klinghammer, Luigi Lorini, Daan Nevens, Christian Simon, Jean-Pascal Machiels, Paolo Bossi

**Affiliations:** ^1^ Department of Hematology, Oncology and Cancer Immunology, Charité—Universitätsmedizin, Berlin, Germany; ^2^ Charité Comprehensive Cancer Center, Berlin, Germany; ^3^ Medical Oncology Unit, Department of Medical and Surgical Specialties, Radiological Sciences and Public Health, ASST Spedali Civili of Brescia, University of Brescia, Brescia, Italy; ^4^ Iridium Netwerk, Radiation Oncology Department, University of Antwerp, Antwerp, Belgium; ^5^ Department of Otolaryngology, Head and Neck Surgery, University of Lausanne, CHUV, Lausanne, Switzerland; ^6^ Institut Roi Albert II, Department of Medical Oncology, Cliniques Universitaires Saint-Luc and Institut de Recherche Clinique et Expérimentale (Pole MIRO), UC Louvain, Brussels, Belgium

**Keywords:** head and neck squamous cell carcinoma, PD-L1 CPS, treatment stratification, survey, HNSCC

## Abstract

Multiple factors differentially influence treatment decisions in the first line treatment of recurrent/metastatic HNSCC. The EORTC Young investigator group launched a survey among treating physicians to explore the main influencing factors for treatment stratification. The questionnaire was posted as a web-survey link from May to August 2020. Next to defining the factors that mostly influence therapeutic decision the survey was complemented by a clinical case discussion of five patient cases. A total of 118 responses from 19 countries were collected. The key factors identified to guide treatment decision were performance status, PD-L1 Expression, time from last systemic treatment above or below 6 months, and disease burden.

Prospective evaluation of patient characteristics and additional potential predictive biomarkers for novel treatment options remains an important question to stratify personalized treatment for RM HNSCC.

## Introduction

Head and neck squamous cell carcinoma (HNSCC) represents the sixth most common type of cancer with 0.65 million new cases and 0.33 million deaths annually worldwide (1). Despite recent advances in the diagnosis and treatment of HNSCC, the median survival for patients with incurable, recurrent, or metastatic disease remains poor at around 10–15 months (2). Treatment intensification failed to improve outcome (3). To date, the epidermal growth factor receptor (EGFR)-targeted antibody cetuximab and programmed death receptor-1 (PD-1) antibodies nivolumab and pembrolizumab are approved as targeted agents for the treatment of HNSCC. With the introduction of immunotherapies both in first and second line treatments of HNSCC, the therapeutic options for patients have increased (4). Toxicities and QoL were favorable with immunotherapeutic treatment in comparison to chemotherapy (5). However, not all patients respond to PD-1 inhibition and for some patients with autoimmune diseases the risk of deterioration with such treatment modalities is essential. Multiple factors have been discussed, which differentially influence treatment decisions in the first line treatment of recurrent/metastatic HNSCC. However there is a lack of scientific evidence to provide adequate patient selection for tailored treatment (6, 7).

The EORTC young investigator group launched a survey among physicians treating head and neck cancer patients, to ask what are the main influencing factors used to stratify treatment for chemotherapy and cetuximab versus immunotherapy alone versus immunotherapy in combination with chemotherapy. Furthermore, we asked the participants to make treatment decisions for particular case presentation taking PD-L1 expression into consideration.

## Methods

The questionnaire was posted as a web-survey link in the EORTC (European Organization for Research and Treatment of Cancer) head and neck cancer (HNC) mailing list reaching 419 EORTC members from May to August 2020. The questionnaire can be found in the **Appendix**. Data were collected *via* Survey Monkey (www.surveymonkey.com) and descriptive analyses were performed.

The survey consisted of 17 items divided in two parts: 12 items in part one and five clinical cases in part two. The participants were medical oncologists, radiation oncologists, and surgeons (otolaryngologist and maxillofacial).

In the first part of the survey we evaluated the experience in treating HNSCC and the environment where each respondent worked (presence or not of a multidisciplinary team). This was followed by the key questions regarding the factors that mostly influence therapeutic decision in the recurrent/metastatic (R/M) setting to choose chemotherapy plus anti-EGFR agent or chemotherapy in combination with immunotherapy (IO) or IO alone. Items to choose were burden of disease, time from last systemic treatment, presence of locoregional or metastatic disease, performance status (PS), tumor pain, hypercalcemia, treatment schedule, risk of bleeding, patient age, PD-L1 combined positivity score (CPS), and the presence of caregiver.

In the second part we proposed five clinical cases with different characteristics and asked the preferred treatment based on three different PD-L1 CPS value (PD-L1 CPS <1; PD-L1 CPS 1–19; PD-L1 CPS ≥20).

## Results

### Collection of Questionnaires

There were 118 responses to the questionnaire. The participants were predominantly male (61%), 53.9% of responders had an age between 40 and 55 years. The majority of participants (43.6%) treated 6–15 HNC patients per month. Countries that mainly contributed to the survey included Italy (40%), Germany (19%), France (6%), Netherlands (6%) Switzerland (6%), and Belgium (6%). Participants were medical oncologists (58.6%), radiation oncologists (20.7%), and head and neck surgeons (20.7%). Of these participants, 43.6% had more than 15 years of experience in head and neck cancer treatment and more than 95% of responders reported to work as part of an HNC multidisciplinary team in their hospital that discusses patients with both curative and palliative intent. Regarding the reimbursement policy for drugs used in R/M HNSCC, in the majority of the countries of the participants, cetuximab in first line only with cisplatin in combination with 5-fluorouracil (67.5%) and nivolumab in platinum resistant patients (92.1%) are reimbursed. Descriptive data of the responders are provided in **Table 1**.

**Table 1:** Participants characteristics.

Participants number (118)	Characteristics	Number (%)
**Gender**	Female	46 (38.9)
	Male	77 (61)
**Age (years)**	<40	39 (33.3)
	40–55	65 (53.8)
	>55	15 (12.8)
	No response	1
**Specialty**	Medical Oncology	68 (58.6)
	Radiation Oncology	24 (20.7)
	Otolaryngologist (ENT)/Maxillo Facial Surgeon	24 (20.7)
	No respone	2
**Years of experience in treatment of HN** **Cancer (years)**	<5	21 (17.9)
	5–15	45 (38.4)
	>15	51 (43.5)
	No response	2
**New RM/HNSCC seen per month in each center**	1–5	34 (29.1)
	6-15	51 (43.6)
	>15	32 (27.4)
	No response	1
**Presence of HN multidisciplinary team in each center**	Yes	114 (97.4)
	No	3 (2.6)
	No response	1
**Type of RM/HNSCC cases discussed in** **HN multidisciplinary team**	All cases with curative intent	6 (5.2)
	All cases with curative and palliative	103 (88.8)
	intent	7 (6)
	Not all cases discussed	2
	No response	
**Reimbursed policy in each country for** **specific drug**	Cetuximab in first line	
	Yes	82 (75.2)
	No	27 (24.8)
	No response	9
	Cetuximab in second line	
	Yes	60 (57.1)
	No	45 (42.9)
	No response	13
	Cetuximab only with Cisplatin +	
	5-Fluorouracil chemotherapy	
	Yes No No response	75 (67.6)36 (32.4)7
	Pembrolizumab in first line	
	Yes No No response	52 (47.3)58 (52.7)8

HN, head and neck; RM HNSCC, recurrent metastatic head and neck squamous cell carcinoma.

### Factors Mostly Influencing Treatment Decisions

Within the questionnaire, physicians were asked to rate 5 factors to stratify the treatment for platinum in combination with cetuximab (e.g., EXTREME or TPEX protocol) in first-line treatment of R/M HNSCC (8, 9). The main factors identified were performance status, time from last systemic treatment >6 months, and a high burden of disease, respectively 82.8, 64.6, and 61.2% of responders. Moreover, we asked the main factors leading to choosing immunotherapy as monotherapy in the same setting, which were performance status (72.2%), PD-L1 CPS ≥20 (72.2%), and time from last systemic treatment <6 months (53.9%).

In the setting of combined treatment with chemotherapy and immunotherapy, physicians guided their decision on performance status and burden of disease (both 69.8%) and PD-L1 CPS 1–19 (62.2%). In **Table 2** factors that mainly influenced treatment decision are summarized.

**Table 2:** Factors that mostly influence treatment decision.

Immunotherapy alone		Immunotherapy + Chemotherapy		Combination of Platinum + Cetuximab	
Characteristics	% of participants	Characteristics	% of participants	Characteristics	% of participants
Perfomance status ECOG	72.7%	Perfomance status ECOG	69.8%	Perfomance status ECOG	82.8%
PD-L1 CPS ≥20	72.2%	High burden of disease	69.8%	Time from last systemic treatment > or ≤6 months	64.7%
Time from last systemic treatment <6 months	53.9%	PD-L1 CPS 1-19	62.3%	High burden of disease	61.2%
Low burden of disease	48.7%	Time from last systemic treatment <6 months	44.3%	Age	44%
Presence of metastatic disease	40.9%	Presence of metastatic disease	41.5%	PD-L1 CPS <1	40.5%
Age	33.9%	Presence of locoregional relapse	34%	Presence of metastatic disease	36.2%
PD-L1 CPS 1-19	27.8%	PD-L1 CPS ≥20	33%	Presence of locoregional relapse	33.6%
Patients wish	26.1%	Tumor pain	33%	Tumor pain	32.8%
Presence of locoregional relapse	25.2%	Age	28.3%	Patients wish	22.4%
Treatment schedule	14%	Presence of caregiver	17%	Risk of bleeding	13.8%
Tumor pain	10.4%	Risk of bleeding	15.1%	Presence of caregiver	13.8%
Presence of caregiver	7.8%	Patient’s wish	8.5%	Treatment schedule	10.3%
Risk of bleeding	5.2%	Treatment schedule	6.6%	Hypercalcemia	3.5%
Hypercalcemia	0.9%	Hypercalcemia	1.9%		

### Clinical Case Discussion

In our survey we proposed 5 different clinical cases in different settings of R/M HNSCC according to pain, extension of disease, comorbidity, presence of caregiver, performance status, and time from last systemic treatment. We asked the participants to choose the preferred therapy (between chemotherapy + cetuximab, IO alone or IO + chemotherapy) regarding three alternative PD-L1 CPS (<1; 1–19; ≥20).

The first clinical case illustrated a male patient of 58 years old with an ECOG of 0 and with history of hypertension and previous hepatitis B (30 years ago). He was a previous smoker (20 packs/years). He consulted an Otorhinolaryngology specialist for having moderate dysphagia. He was diagnosed with an ulcerated lesion at the base of the tongue and right tonsil, and 4–5 cm nodes on the right neck (level IIa). The subsequent magnetic resonance imaging (MRI) and fluorodeoxyglucose–positron emission tomography (FDG-PET) confirmed the presence of an ulcerated right oropharyngeal lesion, pathologic nodes and showed bilateral lung nodules. Final clinical staging was cT4aN2bM1. In **Table 3**, we report the decisions based on different PD-L1 CPS values from the responders.

**Table 3:** Clinical Case 1—Preferred therapy according to CPS value.

	IO alone (%)	IO+ chemotherapy (%)	Chemotherapy + cetuximab (%)
**CPS <1**	2 (1.9)	12 (11.9)	**87 (86.1)**
**CPS 1–19**	7 (7)	**79** (79)	14 (14)
**CPS ≥20**	**46 (46)**	43 (43)	11 (11)

Most frequent answers in the different PD-L1 CPS groups are depicted in bold.

The second clinical case described a male patient of 62 years old diagnosed with HNSCC. He was a current smoker (120 packs/year) without relevant comorbidities, ECOG 0, who underwent a total laryngectomy + right (IIa–IIb–III,V) and left (II–III–IV) selective neck dissection (SND), right hemithyroidectomy and voice prosthesis placement. Based on TNM VIII edition the tumor was classified as pT3 pN2c cM0, R0, squamous cell carcinoma (SCC) with extracapsular extension (ENE+). The patient received adjuvant radiochemotherapy with cisplatin at a cumulative dose of platinum of 240 mg/m^2^. Two years later a computer tomography (CT) scan showed two lung nodules (10 and 8 mm) in the left upper lobe and another peribronchial nodule (8 mm) in the right lower lobe, with unsuccessful biopsy attempt. FDG-PET revealed next to the known lesion a large mass localized to left side of L5-S1 and left hemisacrum with bone erosion. The patient complained of left lower back pain with impaired ambulation, weight loss (5%) due to anorexia and asthenia. PS ECOG 1. Palliative radiotherapy (20 Gy) on L5-S1 was delivered. In **Table 4**, we report the decisions for systemic treatment of the responders regarding this case based on different PD-L1 CPS value.

**Table 4:** Clinical Case 2—Preferred therapy according to CPS value.

	IO alone (%)	IO+ chemotherapy (%)	Chemotherapy + cetuximab (%)
**CPS <1**	6 (6.5)	8 (8.7)	**78 (84.8)**
**CPS 1–19**	25 (26.8)	**52 (55.9)**	16 (17.2)
**CPS ≥20**	**54 (58.1)**	31 (33.3)	11 (8.6)

Most frequent answers in the different PD-L1 CPS groups are depicted in bold.

The third clinical case described a male patient, 71 years old, PS ECOG 0, never smoker. He had a history of ulcerative colitis that was diagnosed at the age of 54 and treated with mesalazine in the clinical phase of remission. He had a left tonsillectomy with partial excision of the base of tongue plus modified left neck dissection (levels I–IV), with final diagnosis of SCC, p16+ and HPV 16 + pT2 pN2b cM0, R0, ENE+, stage III. This treatment was followed by 3 months of adjuvant concomitant chemoradiotherapy on the neck nodes (66/54 Gy) and cisplatin cumulative dose 260 mg/m^2^. Three months later FDG-PET showed mediastinal and right hilar nodes (dimension 17 × 7 and 22 × 12 mm) and a small nodule at the right lower lung with maximum diameter of 8 mm. Bronchoscopy and sampling of the node confirmed the diagnosis of SCC, p16 positive. The patient had no signs or symptoms and maintained a social life and working activities. **Table 5** summarizes the answers of the responders of the questionnaire.

**Table 5:** Clinical Case 3—Preferred therapy according to CPS value.

	IO alone (%)	IO+ chemotherapy (%)	chemotherapy + cetuximab (%)
**CPS <1**	20 (21.5)	8 (8.6)	**65 (69.9)**
**CPS 1–19**	**47 (50)**	22 (23.4)	25 (26.6)
**CPS ≥20**	**70 (74.5)**	9 (9.6)	15 (15.9)

Most frequent answers in the different PD-L1 CPS groups are depicted in bold.

The fourth clinical case described a female patient of 47 years old, with a PS ECOG 2 due to comorbidities with mild mental impairment, anxiety and depression. A caregiver was present to help her with her everyday needs. Medical history revealed arterial hypertension and polyarticular juvenile idiopathic arthritis since her childhood in treatment with methylprednisolone 4 mg. She underwent a mandibulectomy + maxillectomy + right selective neck dissection. The pathology report revealed a grade 2 SCC of the oral cavity, pT4b pN2b (2/55 ENE-) cM0. She underwent adjuvant concomitant chemoradiotherapy, with RT up to 60/54 Gy, and with a cumulative cisplatin dose of 200 mg/m^2^. After three months, due to the appearance of mild dyspnea, a chest CT scan was performed with evidence of left pleural effusion and progression of disease at the lung and a bone lesion art the sternum. In **Table 6**, we report the treatment decision of our responders, based on different PD-L1 CPS values.

**Table 6:** Clinical Case 4—Preferred therapy according to CPS value.

	IO alone (%)	IO+ chemotherapy (%)	Chemotherapy + cetuximab (%)
**CPS <1**	19 (22.6)	7 (8.3)	**58 (69.1)**
**CPS 1–19**	**39 (43.8)**	20 (22.5)	30 (33.7)
**CPS ≥20**	**51 (56)**	12 (13.2)	28 (30.7)

Most frequent answers in the different PD-L1 CPS groups are depicted in bold.

The fifth clinical case was about a male patient, 74 years old, ECOG 1. In October 2014 he underwent concomitant chemoradiotherapy for a supraglottic laryngeal SCC, cT2 cN2 cM0 (up to 70 Gy) with cumulative cisplatin dose of 300 mg/m^2^. Eleven months later he underwent neck dissection due to nodal relapse. Furthermore, 3 years after initial diagnosis, level V on the left was re-irradiated due to unresectable relapse. Another 2 years later a CT showed vascularized tissue with irregular margins of 32 × 32 mm at the left laterocervical site adjacent to the surgical clips. The patient complained about pain localized to the tumor recurrence. Here we report decisions based on different PD-L1 CPS values from responders (**Table 7**).

**Table 7:** Clinical Case 5—Preferred therapy according to CPS value.

	IO alone (%)	IO+ chemotherapy (%)	Chemotherapy + cetuximab (%)
**CPS <1**	14 (16.3)	8 (9.3)	**64 (74.4)**
**CPS 1–19**	28 (32.2)	**48 (55.2)**	11 (12.6)
**CPS ≥20**	**49 (55.7)**	32 (36.4)	7 (7.9)

Most frequent answers in the different PD-L1 CPS groups are depicted in bold.

## Discussion

Treatment decision in R/M HNSCC remains challenging. No internationally accepted treatment guideline exists to guide the decision-making process. Medical treatment is considered the standard approach in the palliative setting, however for a minority of patients salvage surgery or (re-)irradiation might be an option. The survey was answered by the EORTC members of the Head and Neck cancer group, which represents a community of experts of the field and therefore is not representative for all physicians. CheckMate-141, Keynote-040, and Keynote-48 trials (2, 10, 11) led to the introduction of immune checkpoint inhibitors (ICIs) into the palliative treatment of HNSCC in platinum sensitive or resistant patients, thus providing an alternative to chemotherapy, which is beneficial in regard to tumor control for a subset of patients. However, toxicities which impair quality of life do occur more often with chemotherapy combinations, which has led to the widespread use of immunotherapies. Currently, PD-L1 expression is the only established biomarker to stratify treatment decisions (12). However, a multitude of other factors play a minor or major role in deciding for or against a chemotherapeutic regimen with or without the combination with ICIs. Stzurz and Vermorken in their editorial commentary to Keynote-048 showed the complexity of therapeutic choices in R/M HNSCC, other than PD-L1 CPS values (12). Physicians have to consider multiple variables, namely, biological age (from fitness to frailty), tumor dynamics, and burden of disease. In our survey, the most important decision factor in the palliative setting was the performance status of the patient for the three treatment options given. We interpreted this in the way that good performance status is a fundamental prerequisite for receiving chemotherapy, otherwise this treatment being detrimental for the patient. On the opposite, immunotherapy could be perceived as a treatment with less toxicity, therefore suitable also for patients with lower PS. However, it should be acknowledged that PS is also the strongest predictor of PFS and OS in patients treated with immunotherapy (13). Next to performance status, treatment decision for immunotherapy alone or in combination with chemotherapy is mainly guided by the time from last platinum-based treatment and PD-L1 expression. This is in line with the published data of platinum refractory disease (10, 14) and the inclusion criteria of the Keynote-48 trial (2). Currently, limited data from prospective clinical trials exist regarding the dynamics of response under checkpoint inhibition. Due to the limited overall response rate achieved with immunotherapy alone (less than 20% in the unselected population) (2, 10), the majority of physicians participating in this survey voted for the combination with chemotherapy when a situation of high tumor burden exists. It should be underlined that obtaining response to treatment is crucial in particular when facing disease with high tumor burden and corresponding symptoms (15). The survey was complemented by a clinical case discussion, which took most factors guiding treatment decisions into account. Responses appeared to be guided by PD-L1 CPS as one of the most important factors; however, also in case of high PD-L1 CPS, there was a relatively high quote of respondents choosing combination of chemotherapy plus IO, mainly in cases where the high tumor burden (case #1) or the symptoms (mainly pain) complained by the patients justified the need to achieve tumor response (case #2 and #5). It is interesting to observe that also in the third case responses were guided by PD-L1 CPS expression, even in presence of an autoimmune comorbidity. The anamnesis of ulcerative colitis did not lead the majority of participants of the survey to avoid IO, possibly due to the fact that the autoimmune disease was reported as being under control and without high steroid use. Until recently, no large cases series of patients with inflammatory bowel disease and immune checkpoint inhibition have been published, and patients with active autoimmune diseases have been excluded from most of clinical trials leading to registration of IO in the different cancer sites. Also, the fourth case presented with the comorbidity of a polyarticular juvenile idiopathic arthritis, in active treatment with low steroid dose; the disease-free interval since last platinum dose was 3 months, therefore prompting many respondents to propose the use of IO alone. About one-third of the physicians would have chosen a combination of chemotherapy and cetuximab, considered to be less at risk of toxicities than IO.

Given the obvious limitations of such a survey in general, the collected responses were in line with current treatment recommendations by the European Society of Medical Oncology (ESMO) (6). There is broad consent of treatment with ICI in platinum refractory or nonsymptomatic PD-L1 positive tumor patients. For patients with low or negative PD-L1 expression the choice of the appropriate chemotherapy combination with or without IO has not been defined and especially elderly and frail patients, who do represent a large proportion of patients, remain not adequately represented in trials. This makes general treatment recommendations impossible. The aim of systemic treatment should always be evaluated, if being primarily directed to achieve treatment response or to prolong overall survival. Discussion with the patient and caregivers should also be a central point in treatment choice, evaluating preferences and expectations of the patient. Geriatric assessments or evaluation of the frailty of the patient should be prospectively evaluated to improve patient outcome in this vulnerable population (16). These points highlight a field of missing data in the decision process for the right treatment. Considering all these factors, we support treatment personalization as being crucial in clinical decision making, in a time where different treatments are available for R/M HNSCC patients.

### Conclusion

Immunotherapy has changed the therapeutic landscape in RM HNSCC. Our survey showed how clinical decisions in a real world setting are based mostly on performance status, PD-L1 CPS expression, burden of disease, and time from last systemic treatment. Prospective evaluation of patient characteristics and additional potential predictive biomarkers for novel treatment options remain an important quest to stratify personalized treatment for RM HNSCC.

## Data Availability Statement

The original contributions presented in the study are included in the article/**Supplementary Material**. Further inquiries can be directed to the corresponding author.

## Ethics Statement

Ethical review and approval was not required for the study on human participants in accordance with the local legislation and institutional requirements. The patients/participants provided their written informed consent to participate in this study.

## Author Contributions

KK: Conceptualization, Data curation, Formal analysis, Funding acquisition, Investigation, Methodology, Project administration, Resources, Writing—Original draft, and review & editing. LL: Conceptualization, Data curation, Formal analysis, Investigation, Methodology, Project administration, Resources, Writing—Original draft, and review & editing. DN: Conceptualization, Data curation, Formal analysis, Investigation, Methodology, Project administration, Resources, Writing—Original draft, and review & editing. CS: Supervision, and Writing—Review & editing. J-PM: Supervision, and Writing—Review & editing. PB: Conceptualization, Data curation, Formal analysis, Investigation, Methodology, Project administration, Resources, Writing—Original draft, and review & editing. All authors contributed to the article and approved the submitted version.

## Conflict of Interest

KK: Advisory board participation, invited speaker or conference honoraria from: Merck, Sanofi, Merck Sharp & Dohme, Bristol-Myers Squibb. CS: grants from Roche, grants from Intuitive, personal fees from Pfizer, personal fees from Merck, personal fees from MSD, personal fees from Seattle Genetics, outside the submitted work. J-PM: Advisory board member or speaker with honoraria (managed by my Institution): Pfizer, Roche, Astra/Zeneca, Bayer, Innate, Merck Serono, Boerhinger, BMS, Novartis, Janssen, Incyte, Cue Biopharma, ALX Oncology, iTEOS, eTheRNA. Travel expenses: Amgen, BMS, Pfizer, MSD. Data safety monitoring board with honoraria: Debio, Nanobiotix, Psioxus; Uncompensated advisory role: MSD; EORTC: investigator and study coordinator, H & N group Chair. PB: Advisory board participation or conference honoraria from: Merck, Sanofi, Merck Sharp & Dohme, Sun Pharma, Angelini, Molteni, Bristol-Myers Squibb, GSK.

The remaining authors declare that the research was conducted in the absence of any commercial or financial relationships that could be construed as a potential conflict of interest.

## Publisher’s Note

All claims expressed in this article are solely those of the authors and do not necessarily represent those of their affiliated organizations, or those of the publisher, the editors and the reviewers. Any product that may be evaluated in this article, or claim that may be made by its manufacturer, is not guaranteed or endorsed by the publisher.
