# Supplementary material for: Treatment Stratification in First-Line Recurrent or Metastatic Head and Neck Cancer, on Behalf of the EORTC Young Investigator Head and Neck Cancer Group
Source: Front Oncol. 2022 Jan 27;12:730785. doi: 10.3389/fonc.2022.730785 (PMC8830482; doi:10.3389/fonc.2022.730785)
Supplement: Supplementary file 1 [file DataSheet_1.pdf]

Preliminary Questions

1. Your gender?

- Male
- Female

2. Your Age (years)?

- < 40
- 40 - 55
- > 55

3. In which country do you work?

4. Your specialty?

- Medical Oncology
- Radiation Oncology
- ENT/ Maxillofacial Surgery

5. Years of experience in treatment of HN cancer patients?

- < 5
- 5-15
- > 15

6. How many new RM/HNSCC do you see per month in your centre?

- 1-5
- 6-15
- >15

7. Is there in your centre an HN multidisciplinary team?

- Yes
- No

8. Are all RM/HNSCC cases discussed in multidisciplinary team?

- ◯ Yes, only with curative intent
- ◯ Yes, with curative and palliative intent
- ◯ No

9. Reimbursement policy in your country for RM/HNSCC?

	Yes	No
Cetuximab in first line	◯	◯
Cetuximab in second line	◯	◯
Pembrolizumab in first line	◯	◯
Nivolumab in platinum resistant patients	◯	◯

10. When you choose to use a first line treatment for RM/HNSCC with combination of Platinum + Cetuximab (e.g. TPEx protocol), please select the 5 items that mostly influence your decision for this treatment

- ☐ Time from last systemic treatment > 6months
- ☐ PD-L1 CPS < 1
- ☐ Presence of locoregional relapse
- ☐ Presence of metastatic disease
- ☐ Burden of disease
- ☐ Tumor pain
- ☐ Hypercalcaemia
- ☐ Tumor Bleeding
- ☐ Patient wish
- ☐ Performance status
- ☐ Presence of careviger
- ☐ Treatment schedule (lenght of treatment)
- ☐ Age

11. When you choose to use a first line treatment for RM/HNSCC with IO alone, please select the 5 items that mostly influence your decision for this treatment

- [ ] Time from last systemic treatment < 6months
- [ ] PD-L1 CPS 1 - 19
- [ ] PD-L1 CPS ≥ 20
- [ ] Presence of locoregional relapse
- [ ] Presence of metastatic disease
- [ ] Burden of disease
- [ ] Tumor pain
- [ ] Hypercalcaemia
- [ ] Tumor Bleeding
- [ ] Patient wish
- [ ] Performance status
- [ ] Presence of caregiver
- [ ] Treatment schedule (lenght of treatment)
- [ ] Age

12. When you choose to use a first line treatment for RM/HNSCC with IO + Chemotherapy, please select the 5 items that mostly influence your decision for this treatment

- [ ] Time from last systemic treatment < 6months
- [ ] PD-L1 CPS 1 - 19
- [ ] PD-L1 CPS ≥ 20
- [ ] Presence of locoregional relapse
- [ ] Presence of metastatic disease
- [ ] Burden of disease
- [ ] Tumor pain
- [ ] Hypercalcaemia
- [ ] Tumor Bleeding
- [ ] Patient wish
- [ ] Performance status
- [ ] Presence of careviger
- [ ] Treatment schedule (lenght of treatment)
- [ ] Age

First Clinical Case

Male, 58 years old, PS ECOG = 0.
Comorbidities: hypertension, previous acute B hepatitis (30 years old).
Previous smoker (20 pack/years).
He complains of moderate dysphagia, no weight loss, no pain, moderate asthenia.
ENT visit: ulcerated lesion at the base of the tongue and right tonsil; 4-5 cm nodes on the right neck, level 2a.
Neck MRI: "….Ulcerated right oropharyngeal lesion located between the tonsillar lodge and right tongue base (5x2.7 cm and depth of 2.5 cm)… Pathologic nodes at right 2A and 2B levels(respectively of 4 cm and 9 mm of diameter). Bilateral lungs nodules.."

Fdg PET"…pathological uptake at right oropharyngeal level, and in numerous lung nodulations…"

13. Which therapeutic choices according to PD-L1 CPS?

	IO alone	IO + Chemotherapy	Chemotherapy + Cetuximab
CPS < 1	○	○	○
CPS 1 - 19	○	○	○
CPS ≥ 20	○	○	○

Second Clinical Case

Male, 62 years old. PS ECOG = 0

Comorbidities: Right parotidectomy for pleomorphic adenoma, prostatic hyperplasia. Current smoker (120 pack/years).

30/03/2016: Total laryngectomy + right (2a-2b-3-5) and left (2-3-4) SND + right hemithyroidectomy + voice prosthesis placement. EI: G2 Squamous Cell Carcinoma.
Stage: pT3 pN2c cM0, R0, ENE +.

May-July 2016: Concomitant chemoradiotherapy on neck nodes (64 Gy at right level 2-3 , 56 Gy at right level 4-6 and left level 2-3-4), with Cisplatin total cumulative dose 240 mg/m2.
December 2018 : Neck-Chest CT scan "…Two pulmonary nodules (10 mm and 8 mm) in the left upper lobe anterior segment and another peribronchial nodule (8 mm) in the right lower lobe lateral-basal segment."
Unsuccessful attempt at obtaining enough tissue from a biopsy.
March 2019 Fdg PET "...Pathological 18F-FDG uptakes in left lung hilum and in the two known pulmonary nodules. Intense uptake in a large mass localized to left side of L5-S1 and left hemisacrum with bone erosion. Other osteolytic lesions in left ilium, pubis and ischium…"

Second unsuccessful biopsy attempt.
He complains of left low back pain (background 5-6 NRS, peak 9 NRS) with impaired ambulation. Weight loss (5%) due to anorexia and asthenia. PS 1.
03/04-09/04/2019 Palliative radiotherapy on L5-S1 (20 Gy).

14. Which therapeutic choices according to CPS?

	IO alone	IO + Chemotherapy	Chemotherapy + Cetuximab
CPS < 1	○	○	○
CPS 1 - 19	○	○	○
CPS ≥ 20	○	○	○

Third Clinical Case

Male, 71 years old. PS ECOG = 0
Comorbidities: hypertension, ulcerative colitis diagnosed at the age of 54, treated with mesalazine and now in clinical phase of remission.
Non smoker.
03/2018: left tonsillectomy with partial excision of the base tongue + modified left neck dissection (level 1-4), for SCC, p16 + and HPV 16 +
Stage: pT2 pN2b cM0, R0, ENE +, STAGE III (VII ed);pT2 pN2 cM0, R0, STAGE II (VIII ed)
May-July 2018: Concomitant chemoradiotherapy on neck nodes (66 Gy) and Cisplatin cumulative dose 260 mg/m2. October 2018 Fdg PET: appearance of mediastinal and right hylar nodes, 17x7 mm and 22x12 mm and of a small nodule at right lower lung with maximum diameter 8 mm.
Bronchoscopy and sampling of the node:
squamous cell carcinoma, p16 positive.
He has no signs or symptoms.
He maintains a social life and working activities (engineer).

15. Which therapeutic choices according to CPS?

	IO alone	IO + Chemotherapy	Chemotherapy + Cetuximab
CPS < 1	○	○	○
CPS 1 - 19	○	○	○
CPS ≥ 20	○	○	○

Fourth Clinical Case

Female, 47 years old. PS ECOG = 2

Comorbidities: Polyarticular juvenile idiopathic arthritis, in treatment with methylprednisolone 4 mg ; arterial hypertension. Non smoker.

September 2019 Mandibulectomy + Maxillectomy + right SND: G2 SCC oral cavity pT4b pN2b (2/55 ENE-) cM0.

November-December 2019: concomitant chemoradiotherapy 54 Gy on T and N bilateral (1b-4b) with Cisplatin cumulative dose 200 mg/m2.

March 2020: For appearance of dyspnea she performed a CT SCAN: left pleural effusion; progression of disease at lung and sternum, complete response on T.

Frail patient (PS ECOG = 2; anxiety and depression); no pain; presence of caregiver.

16. Which therapeutic choices according to CPS?

	IO alone	IO + Chemotherapy	Chemotherapy + Cetuximab
CPS < 1	○	○	○
CPS 1 - 19	○	○	○
CPS $\geq$ 20	○	○	○

Fifth Clinical Case

Male, 74 years old. PS ECOG = 1
Comorbidities: hypertension; previous smoker (50 pack/years)
10/2014: Concomitant chemoradiotherapy (with Cisplatin) for supraglottic laryngeal carcinoma cT2 cN2 cM0 (70 Gy on T and N) with Cisplatin cumulative dose 300 mg/m2.
07/2015: Left neck dissection due to nodal relapse of disease
10/2017: non surgically treatable nodal relapse, reirradiation on 5 left level
05/2019: Neck Chest CT Scan: "…vascularized tissue with irregular margins of 32 x 32 mm at the left laterocervical site adjacent to the surgical clips, extended to the skin plane. Suspected progression of disease at base of the tongue and pre thyroid neck fat tissue…stable millimetric lung nodules…"

Patient symptomatic and with critical social condition (no relatives)

17. Which therapeutic choices according to CPS?

	IO alone	IO + Chemotherapy	Chemotherapy + Cetuximab
CPS < 1	○	○	○
CPS 1 - 19	○	○	○
CPS > 19	○	○	○
